# The Role of Short Journey Transportation in the Spreading of Swine Pathogens and Antimicrobial‐Resistant Bacteria

**DOI:** 10.1155/tbed/5600771

**Published:** 2026-02-10

**Authors:** Marta Masserdotti, Nicoletta Formenti, Anna Donneschi, Flavia Guarneri, Federico Scali, Claudia Romeo, Enrico Giacomini, Cristina Bertasio, Maria Beatrice Boniotti, Giovanni Loris Alborali, Camilla Luzzago

**Affiliations:** ^1^ Department of Veterinary Medicine and Animal Sciences, University of Milan, Lodi, Italy, unimi.it; ^2^ Experimental Zooprophylactic Institute of Lombardy and Emilia-Romagna “Bruno Ubertini”, Brescia, Italy; ^3^ Veterinary Practitioner, Brescia, Italy; ^4^ Globe Institute, University of Copenhagen, Copenhagen, Denmark, ku.dk

## Abstract

The transport of live pigs poses a risk to on‐farm biosecurity. Trucks can carry pathogens with significant economic and health impacts, including antimicrobial‐resistant (AMR) bacteria. This study aimed to investigate the microbiological contamination of trucks before and after loading, focusing on AMR bacteria and other major pathogens transmissible through feces. Samples were collected by swabbing the internal surface of disinfected empty trucks at farm entry (“clean”) and after loading (“dirty”) and were tested for total plate count (TPC), specific bacteria, and viruses. *Escherichia coli* isolates were also phenotypically and molecularly tested for the presence of extended‐spectrum β‐lactamase (ESBL), other β‐lactamases (AmpC), and carbapenemase. Bacterial counts (both TPC and Enterobacterales counts) and the probability of testing positive for *E. coli*, ESBL/AmpC‐producing *E. coli*, and Rotavirus A varied significantly depending on the truck condition, being significantly higher in “dirty” than in “clean” trucks. Despite a nonsignificant difference, positivity to Rotavirus B showed the same tendency. Conversely, the truck condition had no effect on Rotavirus C, *Salmonella* spp., porcine reproductive and respiratory syndrome virus (PRRSV), and carbapenemase‐producing *E. coli*, which were detected only in samples collected on “dirty” trucks. Although the positivity rate of most agents in “clean” samples was close to zero, the relatively frequent occurrence of *E. coli* and some rotaviruses highlights the importance of improving sanitization procedures. The detection of ESBL/AmpC‐ and OXA‐48‐like‐producing *E. coli* was of particular concern. These findings confirm the role of trucks in spreading pathogens of concern and AMR, highlighting the importance of effective monitoring and proper sanitization procedures.

## 1. Introduction

The approach of veterinary medicine to animal health has transformed considerably over time, especially in the livestock field, turning more and more to preventing diseases than to treating them [1]. The key element to achieve effective prevention is biosecurity, a system of practices and measures aimed at minimizing the risk of introduction, establishment, and spread of animal infections within and among animal populations [2]. Notably, the persistence of pathogens and opportunistic agents within different animal groups acts as a major driver of cross‐contamination and disease transmission between epidemiological units (e.g., herds and farms). This process can be further facilitated by animal transport. According to the World Organization for Animal Health (WOAH), implementing biosecurity plans is thus pivotal for effective disease prevention within individual farms as well as in whole regions. A low risk of pathogen introduction, persistence, and/or spread within the herd leads in turn to a reduction in the use of drugs, especially antimicrobials [3]. Specific to pig farming, previous studies have identified a clear link between the implementation of biosecurity measures and quantifiable improvements in parameters related to antimicrobial use and production [4, 5]. A key measure to prevent the spread of diseases within a farm is avoiding the mixing of pigs of different ages [6, 7]. This can be achieved either by establishing a rigorous workflow and separation of the groups within the farm or by allocating the groups to different farming sites.

Especially in high‐income countries characterized by more intensive pig production and larger herd sizes, the second solution is generally preferred, and pigs typically follow a path that begins on the birth farm and then transitions to a fattening farm, sometimes including a nursery farm dedicated to caring for weaned piglets. Consequently, transporting animals to and from the farm is a common occurrence [8] and is also one of the most critical hazards for farm biosecurity, as it involves breaching the biosecurity interface and potentially having animals, as well as trucks, or even drivers, carry around pathogens of concern for both animal health (e.g., porcine reproductive and respiratory syndrome virus [PRRSV] [9], porcine epidemic diarrhea virus [PEDV] [10–12], and African swine fever virus [ASFV] [13]) and public health (e.g., *Salmonella* spp. [14], antimicrobial‐resistant [AMR] pathogens [15–17]). Efficient truck cleaning and disinfection procedures are thus crucial to prevent the spread of pathogens from one farm to another, to the abattoir, and to operators. Nevertheless, trucks often show residues of contamination when entering the farm [16]. This is due either to improper execution of the cleaning and disinfection procedures or to protocols that target only some specific pathogens and are thus partially effective. Traditionally, legislative and research efforts in the animal transportation field have been focused mostly on improving welfare conditions rather than sanitary ones, and studies on the role of trucks as a potential threat to on‐farm biosecurity are scarce. Furthermore, the lack of studies and data is even more limited when specifically considering the spread of AMR pathogens, as recently reported by the European Food Safety Authority (EFSA) [18].

The World Health Organization recently published a list of bacteria for which new antimicrobials are urgently needed, particularly highlighting the threat posed by bacteria relevant at the nosocomial level that have become resistant to carbapenems and third‐generation cephalosporins [19]. Among these bacteria are Enterobacterales, which are showing rising levels of AMR [20, 21]. Extended‐spectrum β‐lactamase (ESBL)‐producing Enterobacterales and carbapenemase‐producing Enterobacterales are a significant threat to public health due to their association with increased health costs, hospitalization, and mortality rate [22]. Specifically, *Escherichia coli* is frequently included in AMR monitoring programs worldwide [23] as it is a cross‐species pathogen with a great capacity to accumulate resistance genes, mostly through horizontal gene transfer [24, 25]. There is, therefore, an urgent need to investigate and quantify the impact of animal transportation [18] in the transmission and diffusion of AMR, assessing the role of different risk factors such as the hygiene of the transport vehicle. Based on the above considerations, Enterobacterales are considered a sensitive indicator of ineffective cleaning [26, 27] and can also be used to test for the presence of AMR.

In the present work, we investigated the contamination of pig transport vehicles before and after animal loading procedures to assess the effectiveness of cleaning and disinfection measures to which transport vehicles are commonly subjected. We focused on a panel of high‐impact swine pathogens transmissible through feces selected for their economic significance, persistence in the organic matrix, and consequent feasibility of sampling. Additionally, we investigated ESBL/AmpC‐ and carbapenemase‐producing *E. coli* as indicators to evaluate the general risk of AMR spread associated with animal transportation.

## 2. Materials and Methods

### 2.1. Sampling

To investigate the contamination of animal transport vehicles and assess the efficacy of standard cleaning and disinfection procedures, from March to July 2023, we surveyed trucks used for animal transport in 11 pig farms (three weaning farms, four growing farms, three fattening farms, and one farm that loaded animals both at growing and at fattening stage) located in Lombardy, Northern Italy (farms’ sizes are listed in Table S1). The trucks were employed for short journeys (less than 8 h) to transport pigs from farm to farm or from farm to slaughterhouse. The farms were enrolled in the study based on opportunistic criteria related to logistic constraints, feasibility, and voluntary acceptance to participate by farmers.

For each truck, samples were collected in duplicate at two different timepoints, for a total of four samples. Two samples were collected before loading procedures, after the vehicle had arrived on the farm and undergone the disinfection measures adopted by the company (hereafter, “clean” samples). The other two samples were collected at the end of loading procedures, immediately before the truck left the farm (hereafter, “dirty” samples). The collection of “dirty” samples at this stage was intended to capture the initial level of microbial contamination associated with the on‐farm loading process and potential for subsequent dissemination, while also accounting for the time pigs spent in the truck during loading (~1 h). Sterile gauze swabs were rubbed on the floor and on the lower portion of the interior walls of the truck and/or trailer to cover a total area of ~100 × 100 cm (1 m^2^), avoiding those areas that were still wet from the residual disinfectant from the biosecurity procedures at the entrance of the farm. The disinfectant is usually applied on the external surfaces of the “clean” truck entering the farm either manually, by an operator, or automatically, by a disinfection arch located at the entrance.

The swabs were then placed in pairs in sterile sampling bags, stored at room temperature, and within ≤6 h delivered to the laboratory, where swabs were stored at +4°C until further analyses. A total number of 84 samples (buffer pairs) were collected (Table 1). Of the 84 swabs taken, 78 were subjected to both microbiological and molecular investigations, while the remaining six could only be subjected to bacteriological tests due to a sampling error.

**Table 1:** Number of collected samples by rearing stage.

Rearing stage	Number of farms	Number of samples	Number of trucks
Weaning piglets transported to a nursery or to a wean‐to‐finish barn	3	22	11
Growing pigs transported from a nursery to a finishing barn	5^a^	22	11
Finishing pigs transported to slaughterhouse	4^a^	40	20
Total	11	84	42

^a^A farm was sampled both when loading growing pigs and when loading fattening pigs.

### 2.2. Pathogen Selection

The panel of screened pathogens was selected based on several criteria, including their economic impact on the swine industry, fecal shedding, and transmission via oro‐fecal route. These characteristics were considered suitable for assessing the effectiveness of truck cleaning and disinfection procedures. Specifically, we targeted Enterobacterales (*E. coli*, including ESBL/AmpC‐ and carbapenemase‐producing strains, and *Salmonella* spp.), *Brachyspira* spp., *Lawsonia intracellularis*, PRRSV, PEDV, and Rotavirus A, B, C, and H. Pathogens with high infection prevalence but low morbidity were excluded to focus on agents that serve as more specific indicators of acute biosecurity breaches.

### 2.3. Microbiological Analyses

The total plate count (TPC) was performed manually after incubation at 37°C for 48 h on PCA medium of the −3 dilution, according to internal testing method [28–30]. Such testing method has an upper detection limit of 1.5 × 10^6^ CFU/cm^2^. The quantification of Enterobacterales contamination was performed according to ISO 21528‐2 procedures, and the biochemical confirmation was obtained through the oxidase test. The detection and identification of *Salmonella* spp. strains were performed according to ISO 6579‐1:2017/Amd1:2020 and ISO/TR 6579‐3:2014 procedures.

Concerning *E. coli*, bacteriological examinations were performed by using standard bacteriological cultures [31], while, for ESBL/AmpC‐ and carbapenemase‐producing *E. coli*, the following method was applied: after a pre‐enriching phase with BPW (1:10 ratio) at 37°C for 18–24 h, a first selective incubation at 37°C for 18 h was performed for detecting ESBL/AmpC‐ and carbapenemase‐producing *E. coli* using a solid medium (respectively, McConkey + cefotaxime 1 mg/L at 41 ± 2°C and CHROMID CARBA SMART at 37°C, both for 18–24 h). At the end of this first incubation, the suspected colonies were subcultured on solid McConkey Agar and incubated at 37°C for 18–24 h. A single bacterial colony from each phenotype‐positive sample was resuspended in 250 μL of DNase‐Rnase‐free water, and DNA was extracted by lysis‐boiling (98°C for 10 min) for further molecular characterization. After molecular confirmation, the plates were subjected to antimicrobial susceptibility testing (the panels of tested antimicrobials are listed in Table S2). Minimum inhibitory concentrations (MICs) were determined by broth microdilution, and the strains were classified as “resistant” or “susceptible” according to the epidemiological cutoff values (ECOFFs) recommended by the European Committee on Antimicrobial Susceptibility Testing (EUCAST, https://www.eucast.org). Since *E. coli* isolates were also tested for paromomycin (aminosidine) and sulfisoxazole, and internationally recognized standards for these molecules are unavailable, the classification of the resistance profile was based on the clinical cutoff of the internal test method [32–37], which is ≥32 for paromomycin and ≥512 for sulfisoxazole.

### 2.4. Molecular Analyses

The screening for *Brachyspira* spp., *L. intracellularis*, PRRSV, PEDV, and Rotavirus A, B, C, and H was performed by molecular analysis. In particular, PRRSV screening was performed using a commercial kit (virotype PRRSV RT‐PCR Kit, Indical Bioscience) that allows the detection of PRRSV and its discrimination in European, American, and highly pathogenic American strains. All the other pathogens were searched for by means of in‐house methods. In detail, *Brachyspira hyodysenteriae*, *Brachyspira pilosicoli*, and *L. intracellularis* were searched by real‐time PCR using the kit QuantiNova Probe PCR mastermix (Qiagen) and targeting the nox gene region in *Brachyspira* spp. and the aspA gene in *L. intracellularis* [38, 39]. The reaction for the identification of PEDV allows for the detection of the presence of a nucleic acid region of 111 bp of the S1 gene (spike, region 1) [40].

The performed Rotavirus real‐time RT‐PCR method allows the detection of an RNA portion of the VP6 region specific to the A, B, C, and H groups of rotavirus in pigs. The method is based on the use of two multiplex reactions of one‐step real‐time RT‐PCR: in the first multiplex reaction, specific regions of groups A (98 bp) and C (86 bp) are amplified, and in the second multiplex reaction the specific regions of groups B (89 bp) and H (93 bp) are amplified [41].

The primer sequences used to detect the presence of *B. hyodisenteriae* and *B. pilosicoli*, *L. intracellularis*, PRRSV, PEDV, and Rotavirus A, B, C, and H are listed in Table S3.

Finally, a multiplex‐PCR was used to search for the genes coding for ESBL/AmpC (*bla*_CTX-M_ 593 bp, *bla*_SHV_ 237 bp, *bla*_TEM_ 445 bp, and *bla*_CMY_ 820 bp) [42, 43] and carbapenemase (*bla*_VIM_ 390 bp, *bla*_NDM_ 621 bp, *bla*_KPC_ 798 bp, and *bla*_OXA-48-like_ 438 bp) [44]. We investigated seven out of the nine ESBL/AmpC‐producing *E. coli* isolates and nine out of the 10 carbapenemase‐producing *E. coli* isolates that were detected on the plate. On the remaining *E. coli isolates*, it was not possible to conduct the molecular investigation due to either the inability of the strains to regrow during processing or contamination of the samples.

### 2.5. Statistical Analysis

To assess the effectiveness of cleaning and disinfection protocols, the effect of the independent variable “condition” (i.e., “clean” or ”dirty") on i) the presence/absence of pathogens with an overall positivity rate greater than 10% (i.e., *E. coli*, ESBL/AmpC‐producing *E. coli*, and Rotaviruses A, B, and C) and ii) TPC and Enterobacterales count (ln‐transformed values [ln(x + 1)]) was analyzed through mixed logistic regressions and linear mixed models, respectively. In all models, the productive category (i.e., weaning, growing, or fattening) was included as a covariate, and farm IDs were included as random intercepts to account for different samplings within the same farm. When significant, factors with more than two levels (namely, productive category) were tested post hoc through *t*‐tests on differences of least‐square means, applying Holm correction for multiple comparisons. Results were considered statistically significant when *p*  < 0.05. All the analyses were carried out using the packages *lme4* and emmeans in R (R Core Team 2021) version 4.3.1 (2023–06‐16).

## 3. Results

**Figure 1:**
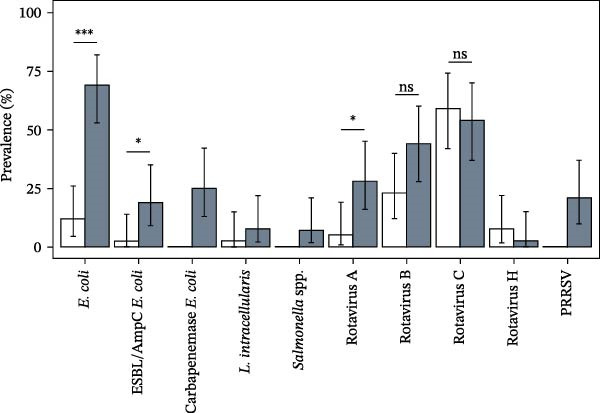
Positivity rate of the examined pathogens in swabs sampled from “clean” (white bars) and “dirty” pig transport trucks (gray bars). Bars indicate 95% confidence limits. When indicated, statistics were performed by linear mixed modeling. In all the other cases, statistical analysis was not performed due to low positivity rate or exclusive occurrence in one condition. *Brachyspira* spp., coronaviruses, and influenza A were never detected. Asterisks indicate statistical significance ( ^∗^ *p* < 0.05 and  ^∗∗∗^ *p* < 0.001), whereas “ns” means “not significant.”.

A total of 84 swabs collected from 42 trucks (42 “dirty” and 42 “clean” swabs) were tested for specific pathogens and bacterial counts. The positivity rates of the pathogens considered and their distribution between swabs from “clean” and “dirty” trucks are reported in Table 2 and Figure 1.

**Table 2:** Overall positivity rate of the investigated pathogens and percentage of positive swabs sampled in “clean” and “dirty” pig transport trucks.

Microorganism	Positivity rate (%)	Percentage on “clean” trucks	Percentage on “dirty” trucks
*E. coli*	40.47% (34/84)	11.90% (5/42)	69.04% (29/42)
ESBL/AmpC‐producing *E. coli*	10.71% (9/84)	2.38% (1/42)	19.04% (8/42)
Carbapenemase‐producing *E. coli*	11.90% (10/84)	0%	23.81% (10/42)
*Salmonella* spp.	3.85% (3/84)	0%	7.14% (3/42)
*Brachyspira* spp.	0%	0%	0%
*L. intracellularis*	5.13% (4/78)	2.56% (1/39)	7.69% (3/39)
PRRSV	10.26% (8/78)	0%	20.51% (8/39)
PEDV	0%	0%	0%
Rotavirus A	16.67% (13/78)	5.13% (2/39)	28.21% (11/39)
Rotavirus B	33.33% (26/78)	23.08% (9/39)	43.59% (17/39)
Rotavirus C	56.41% (44/78)	58.97% (23/39)	53.85% (21/39)
Rotavirus H	5.13% (4/78)	7.69% (3/39)	2.56% (1/39)

For most of the examined pathogens, at least one positive swab was found in both “clean” and “dirty” trucks, with the exception of PRRSV, carbapenemase‐producing *E. coli*, and *Salmonella* spp., which were detected only in “dirty” samples. Regarding the latter, the three isolates were serotyped, resulting in one *Salmonella typhimurium* monophasic variant and two *Salmonella choleraesuis*. No samples positive to *Brachyspira* spp. or PEDV were detected.

Regarding those pathogens detected in both conditions and with an overall positivity rate higher than 10%, the probability of testing positive was significantly higher in “dirty” samples than in “clean” ones for *E. coli* (*p*  < 0.0001), ESBL/AmpC‐producing *E. coli* (*p* = 0.026), and Rotavirus A (*p* = 0.011). Despite a nonsignificant difference (*p* = 0.0504), positivity to Rotavirus B showed the same tendency. The truck condition had conversely no effect on Rotavirus C presence (*p* = 0.10), which varied instead depending on the production stage (*p* = 0.0002), with swabs from weaning and growing farms being more likely to be positive than the ones from fattening farms. Similarly, ESBL/AmpC‐producing *E. coli* were more likely to be found in swabs collected from weaning than fattening farms (*p* = 0.046).

Bacterial counts varied significantly depending on the truck condition as well: Both TPC (*p*  < 0.0001) and Enterobacterales counts (*p*  < 0.0001) were significantly higher in “dirty” samples than in “clean” ones (Figure 2). Complete results of all models, including parameter estimates, are reported in Table S4.

Figure 2Total plate counts (a) and Enterobacterales counts (b) from swabs sampled on the internal surfaces of pig transport trucks at farm entry (“clean condition,” white boxplot) and after loading (“dirty condition,” gray boxplot). Statistics were performed by linear mixed modeling. The upper detection limit of the TPC method is of 1.5 × 10^6^ CFU/cm^2^. Asterisks indicate statistical significance ( ^∗∗∗^ *p* < 0.001).

**Figure:**
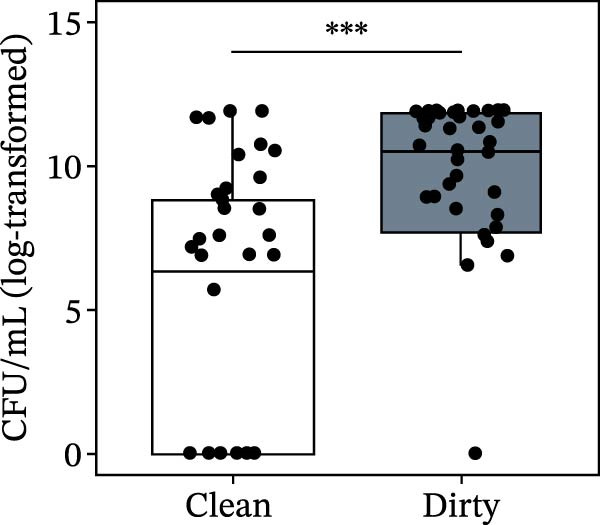
(a)

**Figure:**
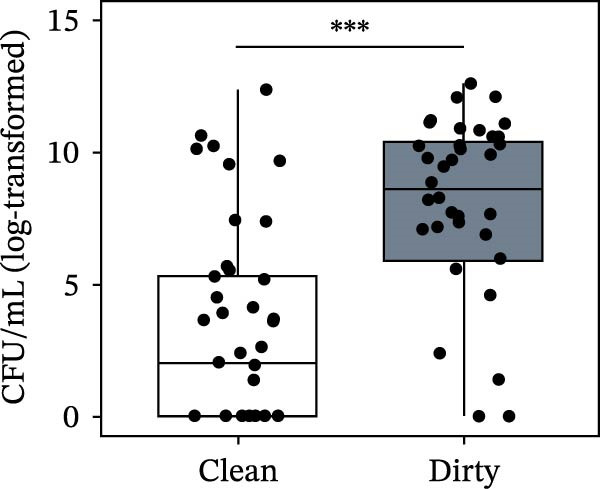
(b)

Regarding AMR bacteria, the proportion of isolates resistant to the different molecules examined and their distribution between “clean” and “dirty” conditions are shown in Table 3 for *E. coli* isolates and in Table 4 for ESBL/AmpC‐producing *E. coli* and carbapenemase‐producing *E. coli*.

**Table 3:** Percentage of resistance of *E. coli* isolates (*n* = 34) for each molecule analyzed and distribution of these percentages between swabs taken on the “clean” and the “dirty” trucks.

Molecule	(Tentative) epidemiological cutoff values (T)ECOFF	Resistance (%)	Percentage on “dirty” trucks	Percentage on “clean” trucks
Ampicillin	8	64.71% (22/34)	90.91% (20/22)	9.09% (2/22)
Tetracycline	8	61.76% (21/34)	85.71% (18/21)	14,29% (3/21)
Florfenicol	16	55.88% (19/34)	89.47% (17/19)	10.53% (2/19)
Trimethoprim + sulfamethoxazole	0.5	50% (17/34)	88.23% (15/17)	11.76% (2/17)
Enrofloxacin	0,125	32.35% (11/34)	90.91% (10/11)	9.09% (1/11)
Gentamicin	2	26.47% (9/34)	100% (9/9)	0%
Amoxicillin + clavulanic acid	(8)	23.53% (8/34)	100% (8/8)	0%
Flumequine	2	23.53% (8/34)	100% (8/8)	0%
Cefazolin	4	20.59% (7/34)	100% (7/7)	0%
Kanamycin	(16)	17.64% (6/34)	100% (6/6)	0%
Colistin	2	2.94% (1/34)	100% (1/1)	0%

*Note:* The epidemiological cutoff is available online (ECOFFs, EUCAST, www.eucast.org, last accessed on February 28th, 2024).

**Table 4:** Percentage of resistance of ESBL/AmpC‐producing *E. coli* isolates (*n* = 9) and carbapenemase‐producing *E. coli* isolates (*n* = 10) for each molecule analyzed and distribution of these percentages between swabs taken on the “clean” and the “dirty” trucks.

Molecule	(Tentative) epidemiological cutoff values (T)ECOFF	ESBL/AmpC‐producing *E. coli*			Carbapenemase‐producing *E. coli*		
		Resistance (%)	Percentage on “dirty” trucks	Percentage on “clean” trucks	Resistance (%)	Percentage on “dirty” trucks	Percentage on “clean” trucks
Cefotaxime	0.25	100% (9/9)	88.89% (8/9)	11,11% (1/9)	80% (8/10)	100% (8/8)	0% (0/8)
Cefepime	0.125	88.89% (8/9)	87.50% (7/8)	12.5% (1/8)	50% (5/10)	100% (5/5)	0% (0/5)
Ceftazidime	1	55.56% (5/9)	100% (5/5)	0% (0/5)	10% (1/10)	100% (1/1)	0% (0/1)
Cefotaxime + Clavulanic acid	0.25	33.33% (3/9)	100% (3/3)	0% (0/3)	60% (6/10)^c^	100% (6/6)	0% (0/6)
Cefoxitin	16	33.33% (3/9)	100% (3/3)	0% (0/3)	10% (1/10)^b^	100% (1/1)	0% (0/1)
Ertapenem	(0.03)	33.33% (3/9)	100% (3/3)	0% (0/3)	100% (10/10)	100% (10/10)	0% (0/10)
Ceftazidime + Clavulanic acid	1	33.33% (3/9)	100% (3/3)	0% (0/3)	10% (1/10)	100% (1/1)	0% (0/1)
Temocillin	16	11.11% (1/9)^a^	100% (1/1)	0% (0/1)	100% (10/10)	100% (10/10)	0% (0/10)
Imipenem	0.5	0%	0%	0%	20% (2/10)^b^	100% (2/2)	0% (0/2)
Meropenem	0.06	0%	0%	0%	80% (8/10)^c^	100% (8/8)	0% (0/8)

*Note:* The epidemiological cutoff is available online (ECOFFs, EUCAST, www.eucast.org, last accessed on February 28th, 2024).

^a^Three samples were classified as susceptible (S) but had values at the cutoff between susceptible and resistant (R).

^b^One sample was classified as S but had a value at the cutoff between S and R.

^c^The remaining samples, classified as S, also had values at the S/R cutoff threshold.

Although they are not listed in Table 3 due to the lack of internationally recognized standards for these molecules, *E. coli* isolates were also tested for paromomycin and sulfisoxazole, and results were interpreted based on the clinical cutoff of the internal test method [31–36]. Around 67.65% (23/34) of the tested *E. coli* samples were resistant to sulfisoxazole. The majority of these (20/23) were collected on “dirty” trucks. Around 14.71% (5/34) of the *E. coli* samples were resistant to aminosidine, all collected on “dirty” trucks.

The seven ESBL/AmpC‐producing *E. coli* isolates that were subjected to molecular investigation by PCR were found to be positive for *bla*_CTX-M_, *bla*_TEM_, and *bla*_CMY_ (alone or in combination), and none was positive for *bla*_SHV_. One isolate was negative to all the sought genes (Table 5). All of the carbapenemase‐producing *E. coli* isolates that were subjected to molecular investigation by PCR were found to be positive only for the *bla*_OXA-48-like_ gene (Table 5).

**Table 5:** Distribution of positivity rate of resistance genes found, either singly or in combination, in ESBL/AmpC‐ and carbapenemase‐producing *E. coli* isolates.

Isolate	*bla*_CTX-M_	*bla*_CTX-M + TEM_	*bla*_CMY + TEM_	*bla*_SHV_
ESBL/AmpC‐producing *E. coli*	42.86% (3/7)	28.67% (2/7)	14.29% (1/7)	0% (0/7)

	*bla*_OXA-48-like_	*bla*_NDM_	*bla*_KPC_	*bla*_VIM_

Carbapenemase‐producing *E. coli*	100% (9/9)	0%	0%	0%

## 4. Discussion

We investigated the potential role played by vehicles for transporting live pigs in the spread of a comprehensive panel of pathogens transmissible through feces, including antibiotic‐resistant pathogens, an aspect that has gaps in knowledge and requires dedicated investigations, as recently reported by EFSA [18].

Overall, sanitized trucks entering the farms exhibited significantly lower bacterial counts and pathogen occurrence compared to loaded trucks, as expected. In particular, several pathogens of zoo‐economic and zoonotic importance, namely carbapenemase‐producing *E. coli*, *Salmonella* spp., and PRRSV, were never detected on “clean” trucks entering the farm. These findings are indicative of the general effectiveness of cleaning and disinfection procedures in reducing potential contamination at farm entry. However, residual contamination by *E. coli*, *L. intracellularis*, and rotaviruses was still observed in some sanitized trucks at farm entry, confirming that existing sanitizing procedures require further improvement. In particular, Rotaviruses B, C, and H were frequently detected on “clean” trucks, with Rotavirus C occurrence being equally likely on “clean” and “dirty” trucks, suggesting that current sanitization protocols might be ineffective against them. In general, rotaviruses are known for their high environmental resistance: drying does not inactivate all viromes [42], and while for Rotavirus A the effectiveness of phenolic disinfectants regardless of the concentration of organic substance has been demonstrated, and glutaraldehyde‐based disinfectants or peroxide compounds have proved to be effective only if preceded by careful cleaning and removal of the organic matrix [43]. Additionally, there is a lack of information regarding the effectiveness of disinfection procedures against Rotavirus C and H, whose potential pathogenicity has only recently been highlighted [7, 41]. The frequent detection of rotaviruses on “clean” trucks provides empirical evidence that current sanitization procedures may be insufficient. Therefore, it is necessary to improve our understanding of the environmental resistance of these pathogens, in order to develop more effective and targeted disinfection protocols toward them.

Studies on the role of trucks as a potential threat to on‐farm biosecurity are usually mainly related to specific outbreak episodes [9, 11, 12] and are thus mostly focused on a single pathogen, not assessing the sanitary risk from a broader epidemiological perspective. For instance, two studies were conducted in our same intensive pig production area in Northern Italy to investigate the role of vehicles as a source of PEDV transmission following the occurrence of an epidemic wave [10] and to assess the propagation of *B. hyodysenteriae* by vehicles for transport to slaughterhouse [44]. Both studies reported relatively high levels of truck contamination, but, interestingly, we did not find any of the two pathogens in our investigation.

The *E. coli* strains detected in this study exhibited resistance patterns associated with the most commonly used antimicrobials in animal farming, particularly against ampicillin (64.7%), tetracycline (61.8%), florfenicol (55.9%), and trimethoprim/sulfamethoxazole (50.0%). Studies that specifically address the detection of AMR bacteria on livestock transport vehicles are limited, which makes comparisons difficult. Nevertheless, these findings are consistent with a recent Italian study on *E. coli* isolated from on‐farm samples collected in an area with production characteristics similar to those in the present study [45], which reported the highest resistance against the same molecules, although at higher rates: ampicillin (95.9%), tetracycline (89.7%), cefazolin (79.3%), and trimethoprim/sulfamethoxazole (74.8%). However, these data should be compared with caution, as the previous study only involved clinical samples [45]. Furthermore, relatively uncommon resistance to last resort antimicrobials, such as broad‐spectrum cephalosporins and carbapenems, aligns with the 2022‐2023 EU summary report on AMR in zoonotic and indicator bacteria from humans, animals, and food. This report highlighted that the prevalence of ESBL‐/AmpC‐/carbapenemase‐producing indicator commensal *E. coli* (and *Salmonella* spp.) collected in the routine monitoring was low during that period [46]. The low levels of colistin resistance observed in this study may be at least partly related to the substantial reduction in colistin usage in Italy, which has decreased by ~99% in recent years [47, 48].

With regard to ESBL/AmpC‐producing strains, our molecular findings are consistent with recent investigations that highlight *bla*_CTX-M_ as the most common gene conferring resistance to beta‐lactams overall [46], detected ubiquitously in community and healthcare contexts, in the environment, in food, and in animal species [49]. For carbapenemase‐producing *E. coli* strains, only *bla*_OXA-48-like_‐encoding strains were found in this study. This gene was reported for the first time in *E. coli* isolated from pigs in 2019 in Germany [50], and it is speculated that transmission occurred from humans, in which this resistance gene was already circulating. Although data on the dissemination of carbapenem‐resistant Enterobacterales (CRE) in livestock are still scarce, a recent study [51] conducted in Italy has shown that OXA‐48‐like‐producing *E. coli* strains, while still relatively uncommon, are consistently being detected with genomic evidence supporting human‐to‐animal transmission routes and are progressively spreading, especially in pigs, at least since 2021. Another recent study reported the detection of *Salmonella enterica* harboring the *bla*_OXA-181_ gene in Italian pigs and pork products [52], further highlighting the ability of OXA‐48‐like carbapenemases to spread across different Enterobacterales species within the food production chain. Considering the importance of carbapenems as last resort antimicrobials for humans, an integrated surveillance approach of CRE emergence is paramount, and the detection of OXA‐48‐like‐producing *E. coli* strains on animal transport vehicles further underlines this aspect. When considering the positivity rate of AMR pathogens across pig production phases, the trend that is observed in the present study is consistent with other studies, where they were more common in early production stages [53]. Overall, the AMR data obtained from trucks in this study are broadly consistent with resistance patterns previously reported in literature, although collected at the farm level through environmental or fecal sampling. While direct comparisons are limited by differences in sampling context and scale, the detection of resistant *E. coli* strains, particularly on outgoing vehicles, underscores the potential role of transport in the dissemination of AMR bacteria. Moreover, although our results do not allow for epidemiological inference, their alignment with broader surveillance studies suggests that animal transport vehicles could serve as strategic sentinel sites within integrated AMR surveillance systems, complementing traditional farm‐level data and helping to assess the risk related to livestock movements in the spread of AMR microorganisms [15, 16]. In general, the high pathogen contamination levels detected on outgoing trucks highlight that ineffective cleaning and disinfection procedures may represent a breach in the biosecurity of the farms visited afterwards. Although animal transport vehicles may play a pivotal role in disseminating pathogens, including AMR bacteria, it is important to emphasize that the control and sanitization of these vehicles should be integrated into a broader strategy that also encompasses the reduction of contamination risks at the source (i.e., on the farm). Consistent application of internal biosecurity measures throughout production cycles is crucial for reducing bacterial carriage, and it has been consistently associated with significant reductions in antimicrobial usage (AMU) on pig farms [54, 55]. Direct measures such as isolation of “stay‐behind” pigs, ensuring proper downtime after sanitization procedures, and the use of slatted flooring in finishing pens seem to effectively reduce the risk of spreading enteric pathogens such as *Salmonella* [56]. Moreover, targeting non‐pig sources of these pathogens via pest control has also been proven to be an effective way of further reducing such risk [57]. The monitoring of environmental hygiene indicators, such as adenosine triphosphate (ATP), may support the validation of cleaning procedures and optimize disinfectant application [58]. Indeed, overuse or misuse of these biocides could lead to an increase in AMR bacteria through co‐selection mechanisms. Metagenomic analyses in pig farming environments have revealed the co‐occurrence of antimicrobial and biocide resistance genes [59], and notably, increased macrolide resistance has been reported in farms with high internal biosecurity, where disinfectant usage is likely to be extensive [60].

It must be noted that our study presents some limitations, in particular the limited number of farms that were enrolled based on opportunistic criteria related to logistic constraints and farmers’ agreement to participate. Such convenience sampling implies that our conclusions might not be extended to the broader pig farming population. However, it is worth noting that farmers willing to participate in such studies may be more engaged with biosecurity practices, which could result in a more conservative estimate of the observed contamination levels.

Although the present study did not focus on truck sanitization and pig loading procedures, observing these procedures revealed critical biosecurity weaknesses that require training and awareness among transporters and breeders. These aspects were recently addressed by Articles 11 and 13 of the Animal Health Law (Reg. [EU] 2016/429), and the variability of biosecurity measures undertaken both during pig loading and truck sanitization procedures made the need for improvements in biosecurity education evident. To achieve the highest biosecurity standards and avoid breakdowns in the biosecurity chain, trucks should be allowed to stand for a period of time after passing through disinfection arches, transportation staff should not be involved in animal handling or entering animal housings, disposable personal protective equipment should always be readily available, and transporters should not be allowed to enter the truck’s cockpit after donning personal protective equipment. Additionally, proper documentation of previous truck sanitization procedures should be made available upon farm entry.

On the whole, the lack of provisions, guidelines, or regulations that precisely address the hygiene of means of transport should be highlighted: for instance, in the European Union, Article 125 of Regulation (EU) 2016/429 on the prevention of transport‐related diseases merely recommends that all equipment and means of transport must be subjected to thorough cleaning, disinfection, and pest control measures to eliminate any potential biohazard, and that other biosecurity measures must be taken depending on the risks associated with the transport operations concerned. This regulation has been supplemented but does not contain any specific provisions yet. However, examples of good practices can be found in national emergency plans. For instance, the recent Italian manual on classical and African swine fever [61] provides a detailed list of authorized disinfectants proven to be effective against these viruses, specifying the required concentration and contact times necessary to ensure complete decontamination [61]. Future research utilizing an ad hoc study design and, possibly, an experimental approach would be valuable for assessing targeted cleaning and disinfection protocols, especially against those pathogens for which information on environmental resistance is scarce (e.g., rotaviruses). Ultimately, bridging the gap between regulations and specific science‐based protocols will be essential to mitigating the role of transport in the spread of swine pathogens.

## 5. Conclusions

To the best of our knowledge, this study is among the first to investigate trucks as mechanical vectors of pathogens relevant to pig breeding and public health, including AMR bacteria. Microbiological investigations carried out on disinfected trucks dedicated to animal transport at farm entry highlight the risk of introducing pathogens, showing that the cleaning and disinfection protocols currently in use are only partially effective, particularly against Rotavirus C. On the other hand, the generally lower bacterial counts and pathogen positivity rates on sanitized trucks, particularly the low occurrence of AMR bacteria in this subset, represent encouraging indicators of the effectiveness of certain cleaning and disinfection procedures. However, given the well‐documented high prevalence of AMR bacteria in livestock systems, this finding should be interpreted cautiously and within the specific scope of our study. Further studies will have to evaluate the effectiveness of specific protocols on farms and especially in farrowing and weaning units. Overall, the variability in sanitization outcomes observed in the present study suggests that there is a fair focus on cleaning and disinfection procedures in animal transport but, at the same time, highlights the need for further studies specifically designed to test the efficacy of different protocols against specific pathogens under field conditions. The definition of guidelines specifying disinfectant classes with proven, targeted biocidal activity along with strict adherence to technical data sheets would allow for greater uniformity in sanitization procedures.

Finally, raising greater awareness and providing targeted training to transporters and farm operators are pivotal to ensuring that protocols are carried out correctly and achieve maximum effectiveness.

## Funding

This work was supported by the Ministero della Salute (Agreement ClassyFarm 2021–2023). Open access publishing facilitated by Universita degli Studi di Milano, as part of the Wiley ‐ CRUI‐CARE agreement.

## Disclosure

A preprint has previously been published [62]. The funder had no role in the design, analysis, and reporting of the study.

## Ethics Statement

Ethical approval was not required according to national and institutional guidelines.

## Conflicts of Interest

The authors declare no conflicts of interest.

## Supporting Information

Additional supporting information can be found online in the Supporting Information section.

## Supporting information


**Supporting Information** The Additional File (.wrd) contains. Table S1: Consistency of the enrolled farms. Table S2: Panel of antimicrobials that were tested for each pathogen. Table S3: Primers and sequences to detect *Brachyspira* spp., *L. intracellularis*, Porcine Epidemic Diarrhea Coronavirus and Rotavirus A, B, C, H. Table S4: Results of mixed logistic regressions and linear mixed models explaining variation in pathogen presence and bacterial counts detected in samples from pig transport trucks before and after loading procedures.

## Data Availability

The data that support the findings of this study are available upon reasonable request from the corresponding author. The data are not publicly available due to privacy restrictions.
