# Supplementary material for: The Role of Short Journey Transportation in the Spreading of Swine Pathogens and Antimicrobial‐Resistant Bacteria
Source: Transbound Emerg Dis. 2026 Feb 10;2026:5600771. doi: 10.1155/tbed/5600771 (PMC12891441; doi:10.1155/tbed/5600771)
Supplement: Supplementary file 1 — Supporting Information The Additional File (.wrd) contains. Table S1: Consistency of the enrolled farms. Table S2: Panel of antimicrobials that were tested for each pathogen. Table S3: Primers and sequences to detect Brachyspira spp., L. intracellularis, Porcine Epidemic Diarrhea Coronavirus and Rotavirus A, B, C, H. Table S4: Results of mixed logistic regressions and linear mixed models explaining variation in pathogen presence and bacterial counts detected in samples from pig transport trucks before and after loading procedures. [file TBED-2026-5600771-s001.docx]

| **Rearing stage**  **(number of sites)** | **Number of samples**  **(number of trucks)** | **Farm size** |
| --- | --- | --- |
| **Weanings** | 22 samples | 600 sows |
| **(3)** | (11 trucks) | 900 sows |
|  |  | 1200 sows |
| **Growings** | 22 samples | 4500 pigs |
| **(5)** | (11 trucks) | 350 sows * |
|  |  | 700 sows |
|  |  | 700 sows |
|  |  | 2500 sows |
| **Fattenings** | 40 samples | 650 sows |
| **(4)** | (20 trucks) | 500 sows |
|  |  | 2000 pigs |
|  |  | 350 sows * |

**Supplementary Table 1**. Consistency of the enrolled farms

*This farm was sampled both when loading growing pigs and fattening pigs.

**Supplementary Table 2.** The panel of antimicrobials that were tested for each pathogen

| ***E. coli*** | **ESBL/AmpC – producing *E. coli***  **OXA-48-like-producing *E. coli*** |
| --- | --- |
| Aminosidine  Amoxicillin + Clavulanic acid  Ampicillin  Cefazolin  Colistin  Enrofloxacin  Florfenicol  Flumequine  Gentamicin  Kanamycin  Sulfisoxazole  Tetracycline  Trimetoprim + Sufametoxazole | Cefepime  Cefotaxime  Cefotaxime + Clavulanic acid  Cefoxitin  Ceftazidime  Ceftazidime + Clavulanic acid  Ertapenem  Imipenem  Meropenem  Temocillin |

**Supplementary Table 3.** Primers and sequences to detect Brachyspira spp., Lawsonia intracellularis, Porcine Epidemic Diarrhoea Coronavirus and Rotavirus A, B, C, H

| ***Brachyspira hyodisenteriae*** | |
| --- | --- |
| **Primer**  **(5’ - 3’)** | RT-Bhyo_FOR: GAC ATG ATG TTA CTA AAA TAG ACT GGG CT |
|  | RT-Bhyo_REV: CAG GCC AAG AAC CAG TAG CAA G |
| **Sonda** | RT-Bhyo_PROBE: (FAM) TTG AAG ACA CTT ACG ATA AAC (MGB) |
| ***Brachyspira pilosicoli*** | |
| **Primer (5’ - 3’)** | RT-Bpil_FOR: GAA GCT ATG CCT AGA GTT ATG GCT AAC |
|  | RT-Bpil_REV: CCT AAA TGC AAT TCT ATA CCA GCA TC |
| **Seq.** | RT-Bpil_PROBE: (FAM) TTT TGA CAA AGA GAT TAC TGA TGA G (MGB) |
| ***Lawsonia intracellularis*** | |
| **Primer (5’ - 3’)** | RT-Law_FOR: TCT CTG CTG CAT GTA ATG AAA TCA |
|  | RT-Law_REV: CTC CTT GAA TAC AAT CCA CAA CAA A |
| **Seq.** | RT-Law_PROBE: (FAM) AAA TGG AGA ACT CCT TGA TC (MGB) |
| **Porcine Epidemic Diarrhoea Coronavirus** | |
| **Primer (5’ - 3’)** | PED_S_FOR: ACG TCC CTT TAC TTT CAA TTC ACA |
|  | PED_S_REV: TAT ACT TGG TAC ACA CAT CCA GAG TCA |
| **Seq.** | PED_S_PROBE: (FAM) TGA GTT GAT TAC TGG CAC GCC TAA ACC AC (BHQ) |
| **Rotavirus A** | |
| **Primer (5’ - 3’)** | Po-RVA_FOR: CAC CTTCAA GAG ARG ATA AYT TRC AA |
|  | Po-RVA_REV: TCG GAT ACC AGG TRK TTA GCCT |
| **Seq.** | Po-RVA_PROBE: (FAM) TCC ATT AGA AGC ATG YTG AT (MGB) |
| **Rotavirus B** | |
| **Primer (5’ - 3’)** | Po-RVB_FOR: TRT GGK GWC ARA ARA TAG CRA T |
|  | Po-RVB_REV: ACC TYT CGA AGC ACT YCC WTT |
| **Seq.** | Po-RVB_PROBE: (VIC) TGA TCC GGC GTC RGC T (MGB) |
| **Rotavirus C** | |
| **Primer (5’ - 3’)** | Po-RVC_FOR: TGT AGC ATG ATT CAC GAA TGG GT |
|  | Po-RVC_REV: ACA TTT CAT CCT CCT GGG GAT C |
| **Seq.** | Po-RVC_PROBE: (CY5) CGC TAG GGG CAA ATG CGC ATG A (BHQ2) |
| **Rotavirus H** | |
| **Primer**  **(5’ - 3’)** | Po-RVH_FOR: CCA CCA CAA YTH GTY CAY TGG TC |
|  | Po-RVH_REV: TCC CAG TGC GTG ACC AGA T |
| **Seq.** | Po-RVH_PROBE: (FAM) GCA TGT TTA ATT GCA GCY TAT TC (MGB) |

**Supplementary Table 4.** Results of mixed logistic regressions and linear mixed models explaining variation in pathogen presence and bacterial counts detected in samples from pig transport trucks before and after loading procedures.

| **Response variable** | **Source of variation** |  | **Parameter estimate±SE** | **Statistic** | **p-value** |
| --- | --- | --- | --- | --- | --- |
| *E. coli* | condition^a^ | dirty | 3.01±0.63 | Χ^2^_1_=22.9 | **<0.0001** |
|  | productive category^b^ | weaner | -1.21±0.71 | Χ^2^_2_=4.1 | 0.13 |
|  |  | grower | -1.21±0.71 |  |  |
| ESBL/AmpC *E. coli* | condition^a^ | dirty | 2.51±1.13 | Χ^2^_1_=4.9 | **0.026** |
|  | productive category^b^ | weaner | 2.20±0.92 | Χ^2^_2_=7.4 | **0.024** |
|  |  | grower | -0.10±1.28 |  |  |
| Rotavirus A | condition^a^ | dirty | 2.26±0.89 | Χ^2^_1_=6.4 | **0.011** |
|  | productive category^b^ | weaner | 1.80±1.03 | Χ^2^_2_=3.3 | 0.19 |
|  |  | grower | 0.45±1.05 |  |  |
| Rotavirus B | condition^a^ | dirty | 1.04±0.53 | Χ^2^_1_=3.8 | 0.050 |
|  | productive category^b^ | weaner | 1.12±0.76 | Χ^2^_2_=2.9 | 0.23 |
|  |  | grower | 0.03±0.73 |  |  |
| Rotavirus C | condition^a^ | dirty | -0.28±0.53 | Χ^2^_1_=2.7 | 0.10 |
|  | productive category^b^ | weaner | 2.73±0.73 | Χ^2^_2_=16.7 | **0.0002** |
|  |  | grower | 1.64±0.59 |  |  |
| TBC | condition^a^ | dirty | 4.00±0.83 | F_1, 79_=22.8 | **<0.0001** |
|  | productive category^b^ | weaner | -0.81±1.90 | F_2, 79_=4.0 | 0.052 |
|  |  | grower | -4.75±1.63 |  |  |
| Enterobacteriaceae count | condition^a^ | dirty | 4.17±0.77 | F_1, 78_=29.2 | **<0.0001** |
|  | productive category^b^ | weaner | 0.34±1.78 | F_2, 78_=1.55 | 0.26 |
|  |  | grower | -2.41±0.77 |  |  |

^a^ clean held as reference level; ^b^ fatteners held as reference level
